# The complete mitochondrial genome of *Ceriagrion fallax* (Odonata: Zygoptera: Coenagrionidae) and phylogenetic analysis

**DOI:** 10.1080/23802359.2020.1870891

**Published:** 2021-02-11

**Authors:** Hainan Shao, Qiurong Li, Yunxiang Liu

**Affiliations:** a State Key Laboratory of Plateau Ecology and Agriculture, Qinghai Academy of Agriculture and Forestry Sciences, Qinghai University, Xining, China; b State Scientific Observing and Experimental Station of Crop Pest in Xining, Ministry of Agriculture, and Provincial Key Laboratory of Agricultural Integrated Pest Management in Qinghai, Xining, China

**Keywords:** Odonata, Coenagrionidae, mitochondrial genome, *Ceriagrion fallax*, phylogenetic analysis

## Abstract

*Ceriagrion fallax* is ubiquitous in south China and is particularly easy be found in some rice fields. In this study, we sequenced and analyzed the complete mitochondrial genome (mitogenome) of *C. fallax*. This mitogenome was 15,350 bp long and encoded 13 protein-coding genes (PCGs), 22 transfer RNA genes (tRNAs) and two ribosomal RNA unit genes (rRNAs). The nucleotide composition of the mitogenome was biased toward A and T, with 74.0% of A + T content (A 42.1%, T 31.9%, C 14.6%, G 11.4%). Gene order was conserved and identical to most other previously sequenced Zygoptera dragonflies. Most PCGs of *C. fallax* have the conventional start codons ATN (seven ATG, two ATT, and two ATC), with the exception of *nad3* and *nad1* (TTG). Except for four PCGs (*cox1*, *cox2*, *cox3*, and *nad5*) end with the incomplete stop codon T––, all other PCGs terminated with the stop codon TAA. Phylogenetic analysis showed that *C. fallax* got together with the same family species (*Agriocnemis femina*, *Enallagma cyathigerum*, *Ischnura elegans*, *Ischnura pumilio*) with high support value. The relationships (Megapodagrionidae + ((Calopterygidae + (Euphaeidae + Pseudolestidae)) + (Coenagrionidae + Platycnemididae))) were supported within Zygoptera.

The insect family Coenagrionidae is placed in the suborder Zygoptera which is known as damselflies. Coenagrionidae is a diverse and ancient group, with the first fossils assignable to this family dated to the early Cretaceous (Chippindale et al. 1999). More than 1100 species are in this family, including six subfamilies: Agriocnemidinae, Argiinae, Coenagrioninae, Ischnurinae, Leptobasinae, and Pseudagrioninae. *Ceriagrion fallax* Ris 1914, one of species within Coenagrionidae, is common in South China. Females and males of this species have different external morphology characters. Here, we sequenced and analyzed the complete mitochondrial genome of *C. fallax*.

Specimens of *C. fallax* were collected from Yongxin County, Jiangxi Province, China (26°54′N, 114°14′E, July 2019) and were stored in Entomological Museum of Qinghai Academy of Agriculture and Forestry Sciences (Accession no. QHAF-CF03). Total genomic DNA was extracted from tissues using DNeasy DNA Extraction kit (Qiagen, Hilden, Germany). A pair-end sequence library was constructed and sequenced using Illumina HiSeq 2500 platform (Illumina, San Diego, CA), with 150 bp pair-end sequencing method. A total of 20.1 million reads were generated and had been deposited in the NCBI Sequence Read Archive (SRA) with accession number SRR12807218. With the mitochondrial genome of *Pseudolestes mirabilis* (FJ606784) employed as reference, raw reads were assembled using MITObim v 1.7 (Hahn et al. 2013). By comparison with the homologous sequences of other Zygoptera species from GenBank, the mitogenome of *C. fallax* was annotated using software GENEIOUS R11 (Biomatters Ltd., Auckland, New Zealand).

The complete mitogenome of *C. fallax* is 15,350 bp in length (GenBank accession no. MW092110), and containing the typical set of 13 protein-coding, two rRNA and 22 tRNA genes, and one non-coding AT-rich region. The nucleotide composition of the mitogenome was biased toward A and T, with 74.0% of A + T content (A 42.1%, T 31.9%, C 14.6%, and G 11.4%). Gene order was conserved and identical to most other previously sequenced Zygoptera dragonflies (Lin et al. 2010; Lorenzo-Carballa et al. 2014; Zhang et al. 2017; Lan et al. 2019; Song et al. 2019). Two rRNA genes (*rrnL* and *rrnS*) locate at *trnL1*/*trnV* and *trnV*/control region, respectively. The lengths of *rrnL* and *rrnS* in *C. fallax* are 1300 and 752 bp, with the AT contents of 77.4% and 75.1%, respectively. All 22 tRNA genes were predicated in this study and vary from 64 bp (*trnC*) to 72 bp (*trnK*). Most PCGs of *C. fallax* have the conventional start codons ATN (seven ATG, two ATT, and two ATC), with the exception of *nad3* and *nad1* (TTG). Except for four PCGs (*cox1*, *cox2*, *cox3,* and *nad5*) end with the incomplete stop codon T−, all other PCGs terminated with the stop codon TAA.

Phylogenetic analysis was performed based on the nucleotide sequences of 13 PCGs from 18 Odonata species. Phylogenetic tree was constructed through raxmlGUI 1.5 (Silvestro and Michalak 2012). Results showed that 17 Zygoptera species divided into three main clades, the new sequenced species *C. fallax* got together with the same family species (*Agriocnemis femina*, *Enallagma cyathigerum*, *Ischnura elegans*, *Ischnura pumilio*) with high support value (Figure 1), indicating the monophyly of Coenagrionidae could be confirmed. Within Zygoptera, the relationships (Megapodagrionidae + ((Calopterygidae + (Euphaeidae + Pseudolestidae)) + (Coenagrionidae + Platycnemididae))) were supported. In conclusion, the mitogenome of *C. fallax* is sequenced in this study and can provide essential and important DNA molecular data for further phylogenetic and evolutionary analysis of Coenagrionidae.

**Figure 1.**
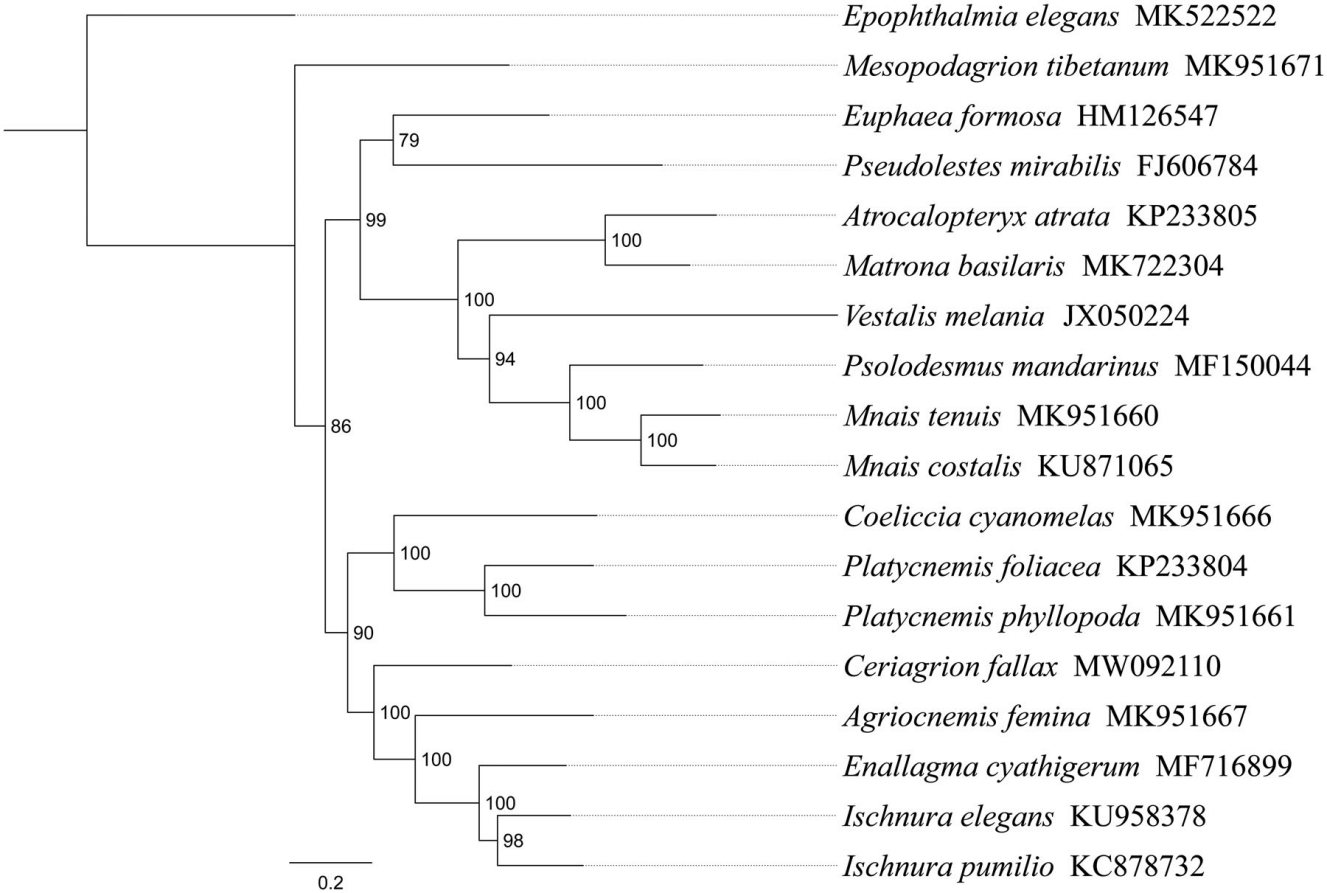
Phylogenetic relationships based on the 13 mitochondrial protein-coding genes sequences inferred from RaxML. Numbers on branches are Bootstrap support values (BS).

## Data Availability

The data that support the findings of this study are openly available in NCBI (National Center for Biotechnology Information) at https://www.ncbi.nlm.nih.gov/, reference number MW092110, SRR12807218.
